# Developing an Intranet-Based Lymphedema Dashboard for Breast Cancer Multidisciplinary Teams: Design Research Study

**DOI:** 10.2196/13188

**Published:** 2020-04-21

**Authors:** Anna Janssen, Candice Donnelly, Judy Kay, Peter Thiem, Aldo Saavedra, Nirmala Pathmanathan, Elisabeth Elder, Phuong Dinh, Masrura Kabir, Kirsten Jackson, Paul Harnett, Tim Shaw

**Affiliations:** 1 Research in Implementation Science and eHealth Group Faculty of Health Sciences The University of Sydney Sydney Australia; 2 Sydney West Translational Cancer Research Centre Sydney Australia; 3 Charles Perkins Centre The University of Sydney Sydney Australia; 4 Faculty of Engineering The University of Sydney Sydney Australia; 5 Sydney Informatics Hub The University of Sydney Sydney Australia; 6 Faculty of Health Sciences The University of Sydney Sydney Australia; 7 Westmead Breast Cancer Institute Sydney Australia; 8 Sydney Medical School The University of Sydney Sydney Australia; 9 Crown Princess Mary Cancer Centre Westmead Hospital Sydney Australia

**Keywords:** eHealth, clinical informatics, human-centered design, data visualization

## Abstract

**Background:**

A large quantity of data is collected during the delivery of cancer care. However, once collected, these data are difficult for health professionals to access to support clinical decision making and performance review. There is a need for innovative tools that make clinical data more accessible to support health professionals in these activities. One approach for providing health professionals with access to clinical data is to create the infrastructure and interface for a clinical dashboard to make data accessible in a timely and relevant manner.

**Objective:**

This study aimed to develop and evaluate 2 prototype dashboards for displaying data on the identification and management of lymphedema.

**Methods:**

The study used a co-design framework to develop 2 prototype dashboards for use by health professionals delivering breast cancer care. The key feature of these dashboards was an approach for visualizing lymphedema patient cohort and individual patient data. This project began with 2 focus group sessions conducted with members of a breast cancer multidisciplinary team (n=33) and a breast cancer consumer (n=1) to establish clinically relevant and appropriate data for presentation and the visualization requirements for a dashboard. A series of fortnightly meetings over 6 months with an Advisory Committee (n=10) occurred to inform and refine the development of a static mock-up dashboard. This mock-up was then presented to representatives of the multidisciplinary team (n=3) to get preliminary feedback about the design and use of such dashboards. Feedback from these presentations was reviewed and used to inform the development of the interactive prototypes. A structured evaluation was conducted on the prototypes, using Think Aloud Protocol and semistructured interviews with representatives of the multidisciplinary team (n=5).

**Results:**

Lymphedema was selected as a clinically relevant area for the prototype dashboards. A qualitative evaluation is reported for 5 health professionals. These participants were selected from 3 specialties: surgery (n=1), radiation oncology (n=2), and occupational therapy (n=2). Participants were able to complete the majority of tasks on the dashboard. Semistructured interview themes were categorized into engagement or enthusiasm for the dashboard, user experience, and data quality and completeness.

**Conclusions:**

Findings from this study constitute the first report of a co-design process for creating a lymphedema dashboard for breast cancer health professionals. Health professionals are interested in the use of data visualization tools to make routinely collected clinical data more accessible. To be used effectively, dashboards need to be reliable and sourced from accurate and comprehensive data sets. While the co-design process used to develop the visualization tool proved effective for designing an individual patient dashboard, the complexity and accessibility of the data required for a cohort dashboard remained a challenge.

## Introduction

### Background

Over the last decade, the quantity of clinical data collected within the health sector has increased exponentially. In parallel, the adoption of digital health, such as electronic health records (EHRs) to collect and aggregate clinical data, has increased. The widespread use of EHRs has the potential to make clinical data more readily accessible to individual health professionals. It also presents opportunities for effective downstream use of clinical data, including quality improvement activities [1], self-directed performance review, personalized professional development [2], and timely clinical research [3].

Despite the proliferation of EHRs, quality and completeness of the data remains a challenge [4,5]. The literature suggests that poorly designed and implemented EHRs contribute to the collection of low-quality clinical data [6]. Another barrier to quality data collection is the lack of interoperability between digital health systems resulting in duplication of data entry and data access issues [7,8], which can result in both cost and workload inefficiencies [9]. Strategies to counteract these barriers, include the redesign of EHR systems to encourage health professionals to enter data at the point of care delivery [10]. Beyond this, there is a need to motivate health professionals to record data consistently and accurately.

One recognized approach to motivate health professionals to collect accurate, high-quality data is to make data visible and useful for clinical practice. However, there is currently a paucity of research on providing health professionals with data in a meaningful way for care delivery. A significant portion of the data entered by health professionals is utilized for mandated reporting and gives limited immediate value to health professionals [4]. The use of clinical dashboards that visually represent such data could provide greater value to health professionals for informing clinical decision making and could also enable performance review [11,12].

Currently, there is no literature on the use of clinical dashboards for data feedback to health professionals specializing in the delivery of breast cancer care. This is surprising given the global burden of breast cancer. In Australia, it is estimated that over 17,210 people will be diagnosed with breast cancer in 2020 [13]. During 2009-2013, individuals diagnosed with breast cancer had a 90% chance of 5-year survival, highlighting the importance of improving the quality of life for patients following breast cancer treatment [13]. In Australia, as in many other countries, breast cancer treatment plans are developed by multidisciplinary teams (MDTs); a team of health professionals, such as breast surgeons, medical oncologists, radiation oncologists, pathologists, radiographers, nurses, and occupational therapists (OTs), together delivers treatment across the care continuum [8].

The research described in this paper focuses on creation of a prototype dashboard for a breast cancer MDT. The specific clinical focus selected for the prototype dashboard was the treatment side effect, lymphedema. This is defined as excess fluid accumulation in a limb causing significant reduction in the quality of life [14]. Of patients treated for breast cancer, approximately 20% will undergo an axillary dissection. Up to 3% of patients undergoing sentinel lymph node biopsy and 10-15% of patients who receive axillary radiotherapy treatment develop lymphedema [15]. The data relevant to this cohort of patients at risk of lymphedema typically come from multiple heterogenous data sources. Therefore, a clinical dashboard that integrates and represents these multiple sources of breast cancer data could assist in the early identification of patients at risk of developing lymphedema, which could significantly improve the quality of life for a large number of people in Australia and globally.

There is little published literature on clinical dashboard use to visualize aggregated data sets to health professionals. A literature review reported how medical dashboards offering health professionals immediate access to critical patient information can improve adherence to quality of care guidelines and may help improve patient outcomes [12]. Another review of the literature indicated that the use of visualization tools in intensive care unit could decrease time spent on gathering data and improve compliance with safety guidelines [16]**.** However, further high-quality detailed research studies are needed to provide evidence of their efficacy and establish guidelines for their design.

### Aims

Dashboards have been effectively used in other industries such as the learning sciences, for feeding back data to both learners and educators to enable more personalized education and training [12-18]. The core of effective design of such dashboards is to follow best practice in user-centered design, including research into user needs, and iterative design and evaluation. The aim of this study was to develop a prototype clinical dashboard for breast cancer MDTs through a co-design methodology and test the prototypes with members of the MDT.

## Methods

### Study Design

The study was informed by a co-design framework [19]**.** This actively engaged end users throughout the project cycle. This process was based on the identification of clinical champions, who shared ownership and support for the methodologies and solutions developed. The study site was the breast cancer department of a major metropolitan hospital in New South Wales, Australia, with a case load of approximately 450 new breast cancer patients per year.

Data integrated and utilized for this study were sourced from routinely collected clinical data sets, including a bespoke breast cancer Structured Query Language EHR for the patient’s administrative treatment and follow-up data and a bioimpedance spectroscopy machine extract for lymphedema data.

Qualitative methods (described in the *Evaluation* section of this paper) were used to evaluate dashboard static mock-ups and the interactive prototypes.

Permission to conduct this study was received from the Western Sydney Local Health District Human Research Ethics Committee.

### Co-Design Process

This exploratory phase of the project aimed to identify methods for improving accessibility of EHR data through visualization platforms such as dashboards. To determine what clinical data were both clinically meaningful and feasible to visualize in a dashboard, 2 focus groups were held. All members of a breast cancer MDT at the study site were invited to the focus groups by the Chair of the MDT. A breast cancer consumer was invited through the National Breast Cancer Foundation. Both focus groups were attended by the consumer representative (n=1) and health professionals (n=33) across a range of disciplines including surgical, medical, and radiation oncology and nursing.

During the first focus group, clinical areas and requirements for data visualization were identified. The aim of the second focus group was to identify an appropriate example clinical area for the focus of a prototype dashboard. MDT members nominated lymphedema as a candidate clinical focus because of its clinical relevance for MDT members and the existing collection of data sources relevant to lymphedema diagnosis and management.

Changes to patients’ lymphedema index (L-Dex) can be monitored using a bioimpedance spectroscopy machine that measures the level of extracellular fluid taken [20]. This L-Dex reading is available to the health professional at the time of the assessment. Health professionals that treat lymphedema, such as OTs, have access to the history of patients’ L-Dex measurements within a paper record. However, OTs have limited access to breast cancer treatment information and rely on patient recall. Conversely, members of the MDT, such as oncologists and nurses, have limited access to L-Dex measurements and treatment information outside of the individual clinician’s specialty.

The translation of the requirements identified in the focus group into a visualization dashboard required close consultation with subject matter experts from the study site. The project team convened an Advisory Committee to oversee the development of the prototype dashboards (Table 1). The Advisory Committee met fortnightly for the duration of the project.

These meetings facilitated building a mutual trust and a shared language between the project team and clinical members. Committee members provided advice on the identification, mapping, and access of relevant data sources, as well as issues surrounding data quality and data completeness and appropriate visualization styles. Committee members also established a set of high-level goals that drove the development of the dashboard interfaces. These were used to create a set of tasks for the evaluation of the prototype dashboard (Table 2). It was noted that some of the tasks pertained to the cohort of breast cancer patients, while others were specific to individual patient data. For this reason, the Advisory Committee decided to create 2 dashboards, 1 to interrogate data of a cohort of patients and 1 to investigate individual patient data.

A 2-step process was used to create and refine the design of the dashboards: (1) static mock-up dashboards were created to gain early feedback on the preliminary dashboard designs and (2) interactive prototype dashboard were created for usability testing. The prototype front end was developed using JavaScript with jQuery, using Store.js for data storage, and Highcharts for information visualization. These tools were considered to be most appropriate for prototype development as they were all well-supported open source libraries that minimized a technical risk for the project.

**Table 1:** Advisory Committee members.

#	Role
1	Breast surgeon
2	Pathologist
3	Medical oncologist
4	Data manager
5	Radiation oncologist
6	5 × project team members (data scientists, implementation scientists, and health service researchers)

**Table 2:** The tasks participants were asked to complete and the completion rate for each task.

ID			Task description		SEQ^a,b^ (median)		Noncomplete tasks
**Individual patient dashboard**							
	1a	You want to understand the procedures this patient has undergone over the course of their treatment. How do you find that out from this dashboard?		2		0	
	1b	Can you please tell me the number of nodes resected in this patient?		1		0	
	1c	Can you please tell me the name of the surgeon that performed the first surgical procedure for this patient?		2		0	
	2a	How do you find that out from this dashboard?		1		0	
	2b	Can you please tell me the BMI^c^ for this patient?		1		0	
	2c	Can you please tell me the date the first L-Dex^d^ reading was taken for this patient?		1		0	
	3a	How do you assess the progress of a patient that has already developed lymphedema from this dashboard?		2		0	
	3b	Can you please tell me whether this patient’s L-Dex readings were on the left or right arm?		1		0	
**Cohort data dashboard^e^**							
	1	You want to identify the proportion of patients with lymphedema that had more than 10 nodes resected? How do you find that out from this dashboard?		1		4	
	2	You want to identify the proportion of patients within the organization that currently have or that have had lymphedema. How do you find that out from this dashboard?		2		0	
	3	You want to identify the proportion of patients within the organization that have no data at all. How do you find that out from this dashboard?		1		0	
	4	You want to identify the proportion of patients within the organization that are having ongoing treatment for lymphedema. How do you find that out from this dashboard?		1		0	
	5a	You want to identify the proportion of patients within the organization that have recovered from lymphedema. How do you find that out from this dashboard?		1		0	
	5b	Can you please tell me how many users these data are based on?		1		0	
	6	You want to identify the proportion of patients within the organization that are having ongoing treatment for lymphedema and have a BMI in the overweight range. How do you find that out from this dashboard?		3		—^f^	

^a^SEQ: Single Ease Question.

^b^Participants (n=5) were asked to rank each task after the completion of a 7-point Single Ease Question scale, where 1=very easy and 7=very hard. No participants rated the tasks as hard or very hard, though as indicated in the table some tasks could not be completed for Dashboard 2.

^c^BMI: body mass index.

^d^L-Dex: lymphedema index.

^e^Participants found it easier to complete tasks on Dashboard 2 as they progressed through the session and became more familiar with the structure of the dashboard.

^f^This task was only completed by 3 participants.

**Figure 1:**
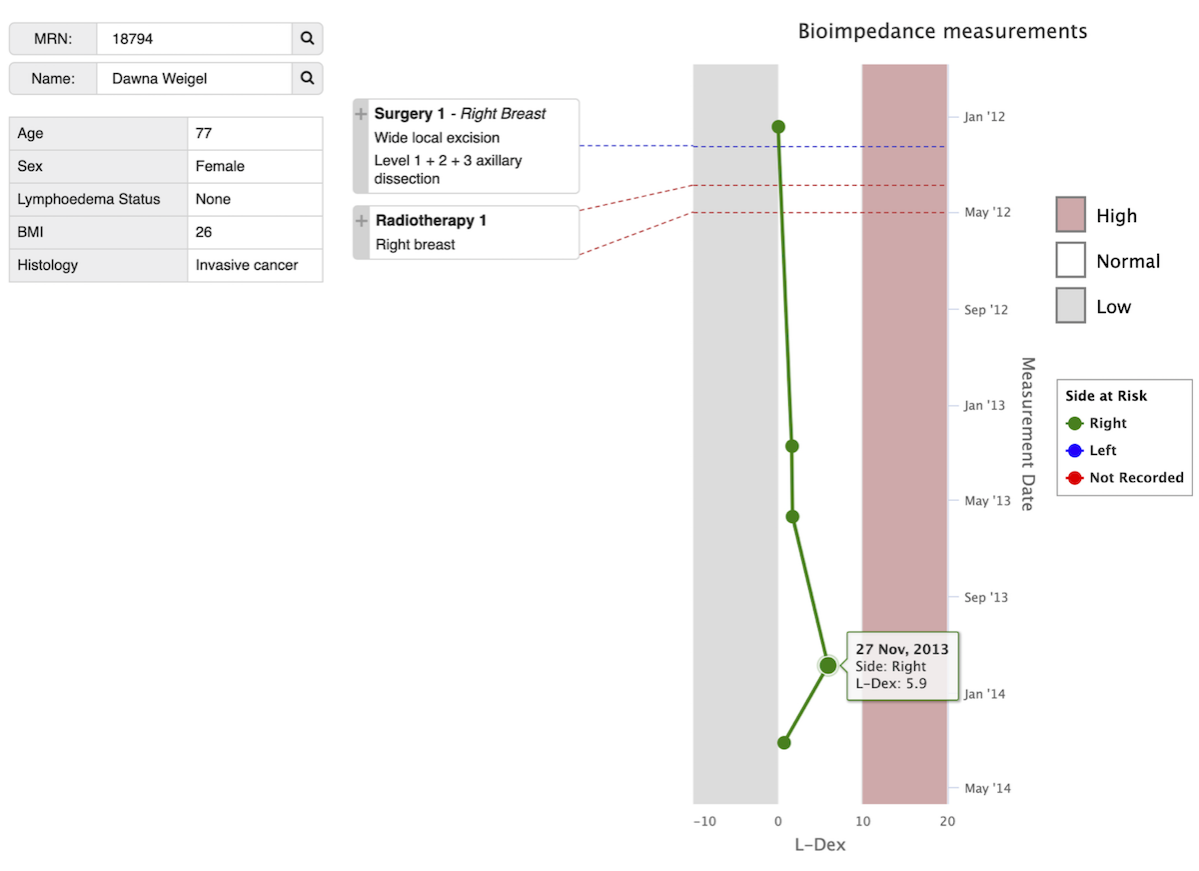
The final prototype of the individual patient dashboard visualizes data for individual patients who have been screened for lymphedema. The prototype dashboard presents a deidentified patient record populated with clinical data. A pseudonym is used for the patient name and medical record number (MRN). This patient has 5 lymphedema index (L-Dex) readings that were taken between January 2012 and April 2014. All the readings are in the normal range for this patient and have been taken on the right side of the body (indicated with green, as opposed to blue for left). The patient had 1 surgery in April. In this figure, the user has clicked on the expand icon (+) next to Surgery 1 to expand the box and see additional details about this procedure. Radiotherapy 1 shows an unexpanded procedure. BMI: body mass index.

**Figure 2:**
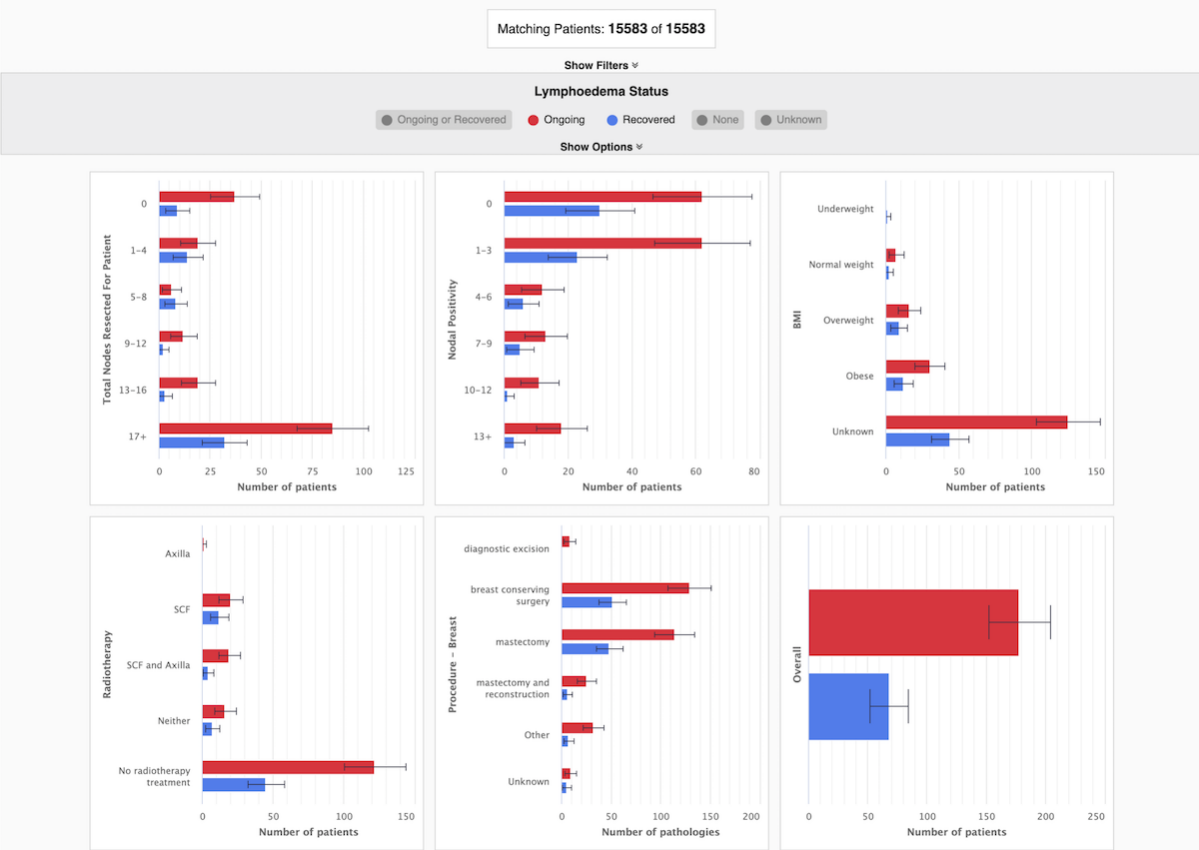
The final prototype of the cohort data dashboard visualizes data for the group of patients who have been screened for lymphedema and presents it to the user in a single dashboard. The dashboard is interactive and by default displays a comprehensive overview of all the cohort data available to the user. In this figure, the dashboard is showing data for all patients who have been diagnosed with lymphedema (indicated in purple) and all patients that have had a lymphedema index (L-Dex) measure &amp;amp;amp;lt;10 (indicated in green). BMI: body mass index.

**Figure 3:**
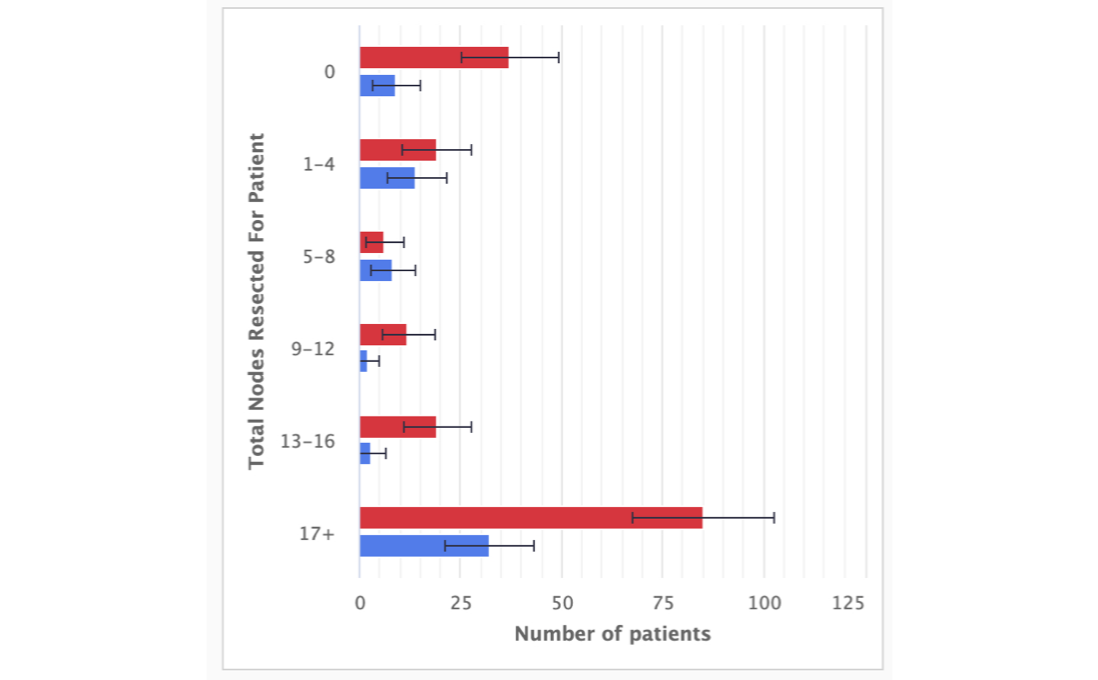
A close-up of one chart on the Cohort Data Dashboard: Nodes Resected.

The static mock-up dashboards (refer to Multimedia Appendix 1 to see Static Mock-up: Individual Patient Dashboard and Cohort Dashboard) were created over a period of 3 months, with feedback sessions as each version was developed for clinicians within the breast MDT (n=3): a breast surgeon, a radiation oncologist, and an OT. In each review session, a facilitator familiar with the dashboards worked face to face with 1 clinician during a 60- to 90-min face-to-face session. During the presentations, each clinician was given an opportunity to provide feedback on the presentation of data and to identify aspects needing improvement. This feedback was reviewed by the development team and informed the next design iteration. This iterative development took 6 months to develop (Figures 1-3). During this time, iterations of the prototype were reviewed by the Advisory Committee.

### Evaluation

The prototype dashboards were evaluated by a purposeful sampling of health professionals (n=5) with expertise supporting breast cancer patients with lymphedema. The evaluation consisted of a Think Aloud Protocol (TAP), where participants worked though the set of concrete tasks (Table 2). After each task, they answered the Single Ease Question (SEQ) [19] with 7-point ranking. The evaluation sessions were conducted by a researcher experienced in the methods used. Participants only had access to the dashboard during the evaluation sessions, as access was provided by the researcher conducting the session. Participants were not paid to participate in the evaluation sessions. The study design made use of TAP for its rich qualitative information about all aspects of use, usability, and experience and the SEQ because it is efficient, which is important for time-poor health professionals. Participants were then asked to complete a semistructured interview to explore their experiences using the dashboards. Each evaluation took between 60 and 90 min to complete.

Data from the SEQ component of the evaluation were aggregated and analyzed by a member of the research team to identify how easy the dashboards were for participants to use. Data from the recording of the semistructured interview component of the evaluation were transcribed and anonymized for evaluation by the research team. A content analysis was undertaken to categorize the transcript data. Categorization of the transcript data was undertaken by a consensus process among 3 researchers. Each transcript was read through by the 3 researchers and line-by-line coding was undertaken to ensure full inclusion of all possible data. Codes were grouped by categories and subcategories comparatively among the 3 researchers until consensus was reached. Exemplar quotations were identified and aligned with relevant categories.

## Results

### Participant Demographics

A total of 5 health professionals participated in the evaluation of the 2 prototypes. Participants were selected from 3 specialties: surgery (1/5, 20%), radiation oncology (2/5, 40%), and occupational therapy (2/5, 40%). Of this cohort, 2 participants had been involved in the evaluation of the static mock-ups. None of the participants had input into the creation of the tasks for the TAP (Table 2).

### Current Clinical Practice

Interviewees consistently noted that the current process for retrieving data on lymphedema patients required access to multiple data sources. This could include multiple electronic databases and digitized or nondigitized clinical notes to find pertinent data about the patient. Interviewees also noted that if data were unavailable from the databases, it was common practice to liaise with another health professional involved in delivering care to the patient to find out additional information. One interviewee highlighted that there are multiple locations which patient data needed to be entered into, cross-referenced, and accessed from:

I have my work emails, I have [the radiation oncology eMR], I have letters I need to review on [the eMR], I have data that I enter in to my own iPad because, I enter data in to our [breast cancer] database...So I feel like I open up a lot of websites just to do my daily job.

BD 1.4

All interviewees stated that they did not currently have regular access to cohort data on lymphedema patients. The only instances when cohort data were available was when an individual actively sought it out, such as during a research project.

### Individual Patient Dashboard

All interviewees were interested and enthusiastic about the individual patient dashboard:

This is brilliant. This is exactly what we wanted when we designed all of this.

BD 1.2

Interviewees did not have easy access to data on how an individual patient progressed through lymphedema screening and breast cancer treatment. The dashboard function in presenting the patient’s treatment journey, in addition to presenting L-Dex measurements over time, was particularly well received:

...it was quite clear and I could see exactly what surgeries they [the patient] had. I didn't have to click on multiple buttons to get there. And you can find out when they had radiotherapy. So a lot here that was clear.

BD 1.3

Interviewees commented positively about color use. For example, the following comment was about red indicating when an individual patient’s L-Dex measurement was moving outside the normal range:

I like this red zone ‘cause for us who don't know the L-Dex exact measurements, it’s good to know. This tells me anything more than 10 presumably is high risk.

BD 1.5

One interviewee commented on the way that patient data were scaled to ensure the user could see the whole time period where data were available. This resulted in different patients’ data displayed across different scales:

The scale is now changed here compared to the other ones. That’s a little bit confusing.

BD 1.1

Multiple interviewees commented that they would have liked additional data incorporated, particularly chemotherapy data (which were not available in the prototype because of medical oncology transitioning to a new information management system at the time of the project). For future deployments, it will be important to support such augmentation. One participant noted:

Yeah, I would have thought that she would have had chemotherapy as well, which doesn’t show at all.

BD 1.1

Overall, interviewees were enthusiastic about the application of the dashboards in clinical practice. In addition, each interviewee identified a range of applications they could utilize the dashboard for, reflecting their diversity of clinical specialties and priorities. Interviewees felt the individual patient dashboard would have particular value as a tool to augment the clinical decision support process. This was because the dashboard provides a means of getting a quick overview of the patient’s pathway through treatment:

It would be for two things, one is to get a quick visualization of what management and what assessment have been done for the patient and, secondly to see the progression.

BD 1.2

In addition, 1 interviewee suggested that the individual patient dashboard may have value to facilitate patient education around their treatment:

I guess for a patient to visualize, sometimes just numbers don’t make a lot of sense to them, but to actually see something on a graph can be helpful.

BD 1.3

### Cohort Data Dashboard

Interviewees were generally excited to have the opportunity to see cohort data on their patients. However, all commented on how much more complex the cohort dashboard was compared with the individual patient dashboard. For example:

I mean, if you’re looking at this [the cohort dashboard], it’s a little bit more complex information than the single patient, because the single patient just hits you without any... You don’t need to work anything out, it just tells you what it is straight away. This one, I needed to get my head around what we were actually looking at.

BD 1.2

Interviewees pointed to the inconsistency in the x-axis in the body mass index graph which is different from the others. Similarly, the y-axis is inconsistent for the 2 graphs about nodes (resected and positivity). Although they saw this as a minor issue, they explained that they wanted to be able to compare information across the charts. One interviewee also noted that having to do calculations of what the charts were saying was a barrier to use:

So I had to highlight a couple of extra things, I suppose the main thing would be I’d have to do a calculation of that, minus one, to the, minus 1.5 up to the 5.9.

BD 1.1

Finally, interviewees commented on instances where terminology used to describe data did not reflect how clinicians routinely conveyed information relating to lymphedema identification and treatment. This issue occurred for both the individual patient dashboard and the cohort data dashboard. Interviewees understood what the terminology used in the prototype dashboards meant, so it was not a barrier to use, merely an area for future improvement. An example of this was:

It’s interesting, just using the word recovered, it’s a tricky one, because they have no clinically overt symptoms but they are considered to be subclinical, like as in their system is perhaps, like they've got ongoing risk and it may actually turn up again, so yeah, recovered just makes it sound like it's…

BD 1.1

As was the case with the individual patient dashboard, interviewees identified issues around the completeness of the clinical data. The quality of the data was viewed as a problem for the long-term usability of the dashboard, even if the interface was user-friendly and engaging. The interviewees frequently drew on their clinical expertise to question the accuracy of the data or identify data points which they felt were missing or did not make sense. There were numerous data points on the cohort dashboard that interviewees did not expect based on their clinical expertise. This was perceived to reduce the level of trust in visualizations, which would limit the likelihood of continued use in clinical practice. The issues could be resolved during the process of refining the prototype for implementation into the clinical setting.

Interviewees suggested that the cohort dashboard would have value as a tool for helping patients understand treatment and lymphedema. Unlike the individual patient dashboard, the cohort data dashboard was viewed as valuable for helping patients understanding the outcomes of the cancer center.

If I see a patient and the patient asks me, “What is your outcome?” Or they want to know, “What’s my survival?” And I say, “Our centre here is excellent and stage three gives you that.” That [the cohort dashboard] might be useful.

BD 1.5

Furthermore, some interviewees indicated that the cohort data dashboard may be useful for research and feedback and to support interaction among different health professionals.

## Discussion

### Principal Findings

The findings of this study demonstrated that it was feasible to use routinely collected data and visualization tools to facilitate clinical decision making and monitor care delivery. This finding builds on the existing literature which has shown that there is considerable interest from health professionals in improving access to routinely collected clinical data [21]. Further, findings from the study do not just demonstrate feasibility of visualizing data but also highlight a number of considerations for designing visualization tools to meet the needs of health professionals in clinical practice.

The individual patient dashboard was successful. Key features of the interface are as follows:

- The side-by-side access to the standard medical record information
- The visualization of the patient trajectory over their full cancer journey

This interface was designed for frequent use by diverse clinicians and the MDT. Our evaluation indicates that it was easy to understand and use. All participants completed all 9 tasks without assistance. The SEQ scores indicate that participants considered all tasks as easy, with median scores for all 1 (very easy) or 2 (easy). User comments point to small refinements but confirm that the overall design is effective.

The cohort data dashboard is far more complex but understandable, with feedback from participants demonstrating enthusiasm about having access to cohort data. The design of this dashboard was driven by the aspirations to understand many dimensions of the data. The design team was aware that visualizing the cohort data was extremely complex but concluded that it would be valuable to gain insights from evaluations at this stage. While 4 of the 5 participants had difficulties with the first task, they were successful in completing the next 6 tasks. The seventh task had high noncompletion rates as it was affected by time pressures on participants. The SEQ scores also indicate perceived high ease of use, with median scores of 1 (very easy) for all but the first 2 tasks, reflecting the start-up learning. Overall, the dashboard is promising for in-depth use by individuals and teams who review these big-picture outcomes infrequently, perhaps months apart. For such intermittent use, we envisage that it may be helpful to add scaffolding to support exploration of key aspects as well as a history mechanism to enable clinicians and administrators to track progress in management and changes.

The study described in this paper revealed that health professionals, seeing the aggregated data for the first time, could identify that there are problems in the underlying data in terms of both its completeness and accuracy. Collecting high-quality clinical data is an acknowledged challenge in the literature [8]. On the one hand, this highlights the need to carefully consider the potential biases in the information displayed. On the other hand, our dashboards have the potential to be a starting point for tackling this problem because both dashboards made data omissions and some errors more visible. They also have the potential to help if they consistently provide value from accurate data so that busy clinicians see value in creating higher-quality records. In instances where data were missing from the prototypes, clinicians made inferences regarding what they expected to have happened to the patient. As highlighted in the evaluation, health professionals drew on their clinical expertise to critically analyze the data presented in visualization tools. A key consideration for future dashboard development is the investment in identifying data sources required to populate the final dashboard and ensuring all relevant sources are incorporated.

The process for developing the dashboards highlighted that we should explore the design of our dashboards for the case of lymphedema in the context of breast cancer. This choice was driven by both the priorities of the health professionals involved in the project and the availability of key data sources, including from medical records and a separate store of lymphedema data. A complex and iterative design process was used to identify the dashboard priorities and refine them to be fit for purpose in the clinical setting. It began with focus group sessions involving 33 members of MDTs and a consumer, then months of fortnightly consultations with a team of 10 to refine the choice of problems, data, and to inform and then refine the design of the dashboards. We believe that we could streamline this for future dashboards, drawing on the lessons from this work. The work described in this article aggregated and made available practice data on lymphedema the first time in the MDT setting. The enthusiasm of the evaluation participants for this information highlights the potential power of such work.

### Limitations

This study is limited due to the small sample size (n=5) used to evaluate the final dashboards. The 5 participants were part of the original focus group meetings due to the commitment of 90 min of clinical time to participate in the evaluation, which may have led to bias in the findings. In addition, the study is limited by the incomplete data set available for developing the prototype dashboards. The data were incomplete as patients received care across different institutions, and data were only available relating to treatment delivered in the organization where the study was conducted. Finally, although participants were undertaking a TAP under artificial conditions, the health professionals were under time pressure to get the tasks done.

Future researchers exploring the use of dashboards for use in health care may wish to explore questions around how users focusing on certain aspects of data such as performance measures may affect their use of all features in a tool. In addition, future research is warranted on the types of data presented in the dashboard and the balance between presenting data that are clinically relevant and data that are easily measurable.

### Conclusions

Health professionals have a considerable level of interest in tools for increasing the accessibility of their routinely collected clinical data using visualization tools. However, there is currently little research into the design of such tools or strategies for implementing them into clinical workflow. Next steps for implementing dashboards into routine clinical practice include the identification of metrics that are highly relevant to clinicians and teams, rather than metrics just easily measurable. In addition, to implement dashboards into practice, it is necessary to not just understand the type of data that has value for presentation in dashboards but investigate when and how they are most useful to health professionals.

A central consideration when designing data visualization tools for health professionals is ensuring they present data in a manner which can be understood and actioned quickly and easily by end users. Furthermore, it is important that iteration is used to review and refine the quality of clinical data being presented to ensure it aligns with the priorities of the health professionals using it. Finally, the creation of visualization tools that meet the needs of health care teams is an interdisciplinary process which requires collaboration between domain experts, data scientists, developers, and user interface designers.

## Appendix

Multimedia Appendix 1 Early mock-ups of the static individual and cohort dashboards that were used to get initial feedback from users.

## Abbreviations

EHR: electronic health record
L-Dex: lymphedema index
MDT: multidisciplinary team
OT: occupational therapist
SEQ: Single Ease Question
TAP: Think Aloud Protocol
