# Supplementary figures and images for: Developing an Intranet-Based Lymphedema Dashboard for Breast Cancer Multidisciplinary Teams: Design Research Study
Source: J Med Internet Res. 2020 Apr 21;22(4):e13188. doi: 10.2196/13188 (PMC7201315; doi:10.2196/13188)

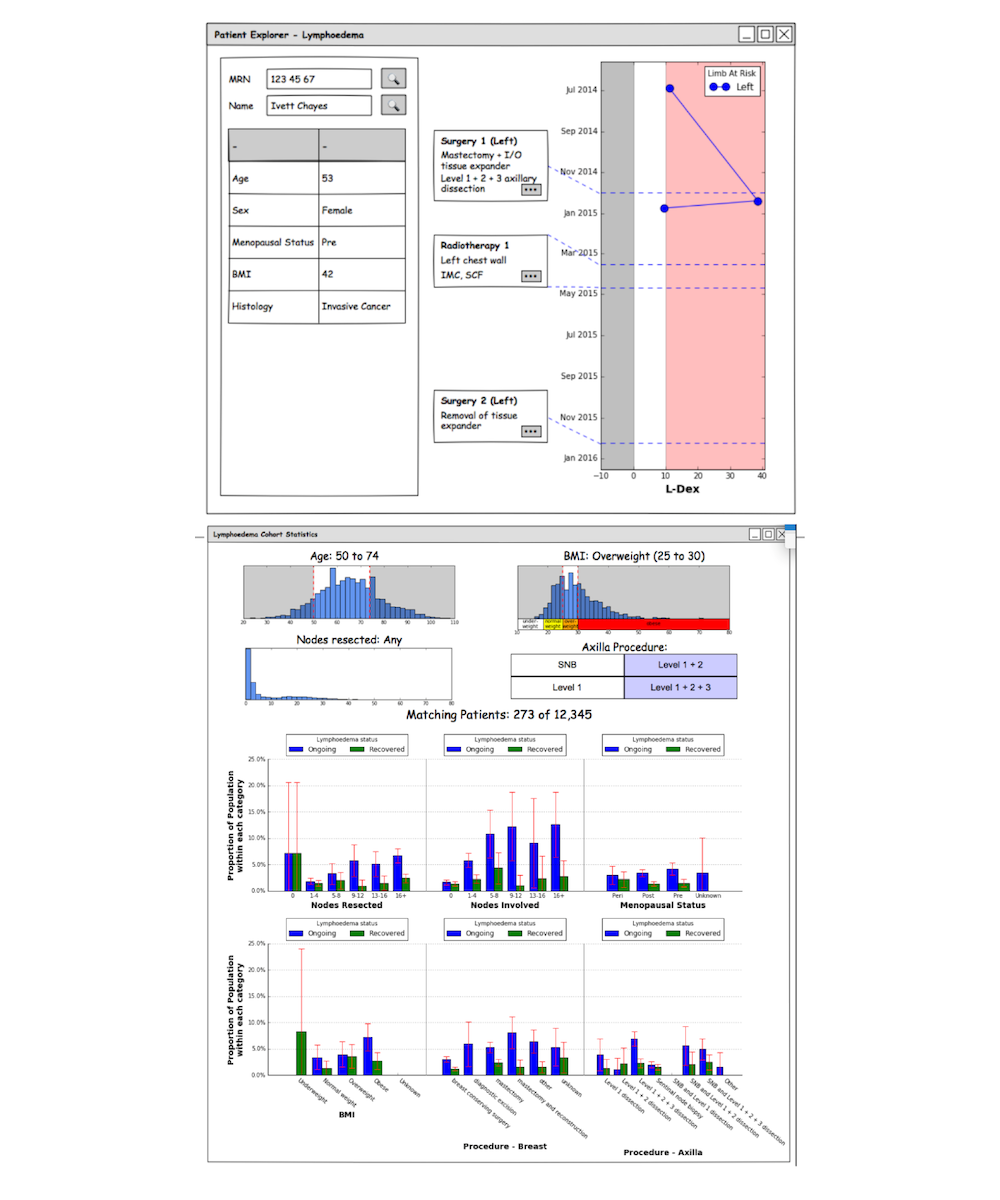

Supplement: Multimedia Appendix 1 [file jmir_v22i4e13188_app1.png]
